# Cumulative use of therapeutic bladder anticholinergics and the risk of dementia in patients with lower urinary tract symptoms: a nationwide 12-year cohort study

**DOI:** 10.1186/s12877-019-1401-y

**Published:** 2019-12-30

**Authors:** Yi-Chi Wang, Yung-Liang Chen, Chun-Che Huang, Chung-Han Ho, Yu-Tung Huang, Ming-Ping Wu, Ming-Jung Ou, Chiu-Hsien Yang, Ping-Jen Chen

**Affiliations:** 1 Department of Family Medicine, Kaohsiung Municipal Hsiao-Kang Hospital, Kaohsiung Medical University, Kaohsiung City, 812 Taiwan; 2 Department of Family Medicine, Kaohsiung Municipal Ta-Tung Hospital, Kaohsiung Medical University, Kaohsiung City, 801 Taiwan; 3 Department of Healthcare Administration, College of Medicine, I-Shou University, Kaohsiung City, 824 Taiwan; 4 Department of Medical Research, Chi-Mei Medical Center, Tainan City, 710 Taiwan; 5 Department of Hospital and Health Care Administration, Chia Nan University of Pharmacy and Science, Tainan City, 717 Taiwan; 6 Center for Big Data Analytics and Statics, Chang Gung Memorial Hospital, Taoyuan City, 333 Taiwan; 7 Division of Urogynecology, Department of Obstetrics and Gynecology, Chi Mei Foundation Hospital, Tainan, Taiwan; 8 Department of Obstetrics and Gynecology, College of Medicine, Fu-Jan Catholic University, New Taipei City, Taiwan; 9 Department of Family Medicine, Kaohsiung Medical University Hospital, Kaohsiung Medical University, Kaohsiung City, 807 Taiwan; 10 School of Medicine, College of Medicine, Kaohsiung Medical University, Kaohsiung City, 807 Taiwan; 11 Marie Curie Palliative Care Research Department, Division of Psychiatry, University College London, London, W1T 7NF UK; 12 Palliative Care Center, Chi-Mei Medical Center, Tainan City, 710 Taiwan

**Keywords:** Anticholinergics, Cohort study, Cumulative defined daily doses, Dementia, Lower urinary tract symptoms

## Abstract

**Background:**

Studies have shown an association between lower urinary tract symptoms (LUTS) and an increased risk of dementia. Whether anticholinergic use contributes to the development of dementia in patients with LUTS remains unknown, especially in Asian populations. This study aims to investigate the association between anticholinergic use and dementia in patients with LUTS.

**Methods:**

This study included patients aged 50 years and over with newly diagnosed LUTS (January 2001 to December 2005), divided into four groups according to their cumulative defined daily doses (cDDDs) of anticholinergics: < 28 cDDDs, 28–84 cDDDs, 85–336 cDDDs, ≥337 cDDDs. Patients were followed up until dementia developed or until the end of 2012.

**Results:**

We recruited a total of 16,412 patients. The incidence of dementia was 10% in the < 28 cDDD group, 8.9% in the 28–84 cDDD group, 11.5% in the 85–336 cDDD group, and 14.4% in the ≥337 cDDD group (*p* = .005). In a Cox proportional hazards analysis, the adjusted hazard ratio of dementia was 1.15 (95% CI = 0.97–1.37) in the 85–336 cDDD group, and 1.40 (95% CI = 1.12–1.75) in the ≥337 cDDD group after adjusting for covariates.

**Conclusions:**

Our study indicates that higher cumulative anticholinergic exposure is associated with an increase in the risk of incident dementia in patients with LUTS aged 50 years of age and over. Either using one anticholinergic agent or switching anticholinergic agents cumulatively increases this risk. Therapeutic risks and benefits of using anticholinergics in LUTS treatment should be clinically reviewed and weighed.

## Background

Lower urinary tract symptoms (LUTS) is a general term for symptoms related to urinary storage and/ or voiding disturbances [1], including frequency, urgency, nocturia, and incontinence. LUTS is a common medical issue in older people [2], and its prevalence and severity have been shown to increase even into the tenth decade of life [3]. LUTS have also been proven to interfere with activities of daily living [2] and are associated with poorer quality of life [4]. The severity of LUTS are a risk factor for sleep disturbance [5], and these have been strongly associated with mental illness, such as depression and anxiety [6, 7]. LUTS have a significant impact on healthcare costs [8], social welfare, and the healthcare system [9, 10].

Dementia is characteristically a disease of older people [11]. Worldwide, 48 million people currently live with dementia; due to the aging population, its prevalence is expected to triple by 2050 [12]. Dementia not only causes disability and death, but also increases the risk of requiring nursing home care [13] and contributes to burden on caregivers [14]. Despite an increasing global disease prevalence, effective therapy to treat dementia is still lacking [15]. To date, many potentially modifiable risk factors for dementia prevention have been proposed, including lifestyle management, vascular risk factor control, and nutritional support [16]; however, the overall evidence is not strong.

In our previous study, we found that LUTS were associated with an increased risk of dementia [17]. However, whether LUTS are a direct risk factor for dementia or whether there are confounding factors remains unknown. Voiding requires a complex mechanism concomitantly regulated by the brain and the urinary system. Evidence suggests that small vessel disease of the brain affects white matter, and white matter disease can cause LUTS, especially in older people [18].

Treatments for LUTS include behavioral therapy, medication, and surgery. Alpha adrenergic-receptor antagonists, 5-alpha reductase inhibitors, anticholinergics, beta 3-adrenoceptor agonists, and phosphodiesterase 5 inhibitors are commonly used medications [19]. Anticholinergics play an important role in treating LUTS by blocking cholinergic muscarinic receptors in the bladder and decreasing involuntary detrusor contractions [20]. However, compelling evidence suggests that anticholinergics are also associated with an increased risk of brain atrophy, dysfunction [21], and dementia [22].

Whether bladder anticholinergic use contributes to increased risk of incident dementia in patients with LUTS is still unclear. Therefore, we used Taiwan’s National Health Insurance Research Database (NHIRD) to conduct a cohort study to investigate our hypothesis that cumulative use of anticholinergics is associated with a higher risk of incident dementia.

## Methods

### Data source

This retrospective, population-based cohort study used data from the Longitudinal Health Insurance Database (LHID), from which 1 million beneficiaries were randomly selected from the NHIRD in Taiwan. There were no differences in age, sex, or average insured payroll-related premiums between the LHID sample and all NHIRD enrollees. Data on outpatient visits, hospital admissions, prescriptions, disease status, and demographics were retrieved from the LHID database. To protect confidentiality all patient and medical institution identification numbers were encrypted and maintained by the National Health Research Institutes of Taiwan before the data were released. The International Classification of Disease, 9th revision, Clinical Modification (ICD-9-CM) coding system was used to classify diagnoses in the LHID. The study was approved by the Institutional Review Board of Chi Mei Medical Center (IRB No. 10708-E01), and the requirement for informed consent was waived.

### Study population

**Fig. 1:**
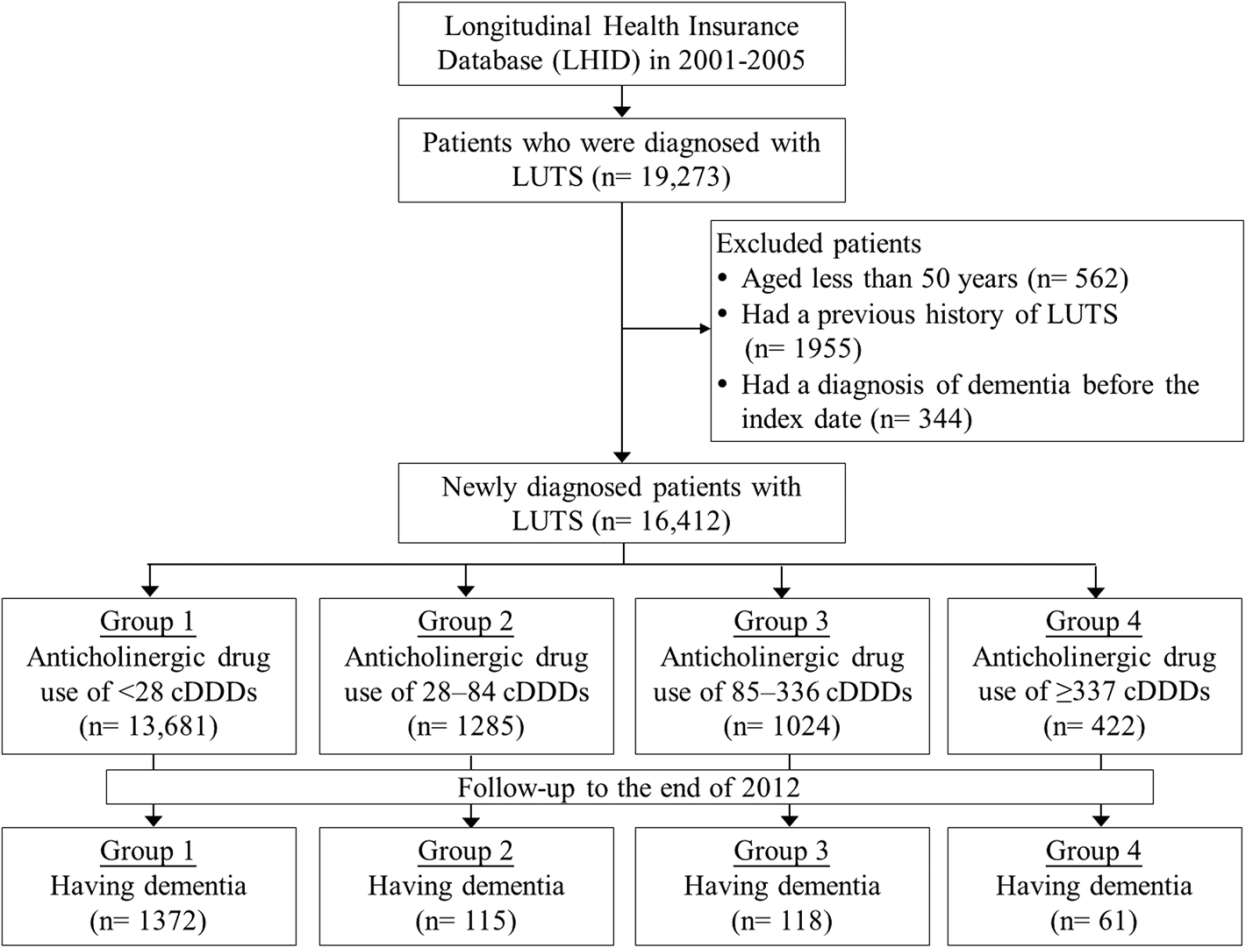
Flow diagram of sample selection

A total of 19,273 patients with at least three outpatient visits or one inpatient admission with a principal diagnosis of LUTS (ICD-9-CM codes 596.51, 600.x, 625.6, 788.2, 788.31–788.33, 788.35, 788.36, 788.4 and 788.6) were identified from January 2001 to December 2005 to validate the accuracy of the diagnoses. We excluded patients aged younger than 50 years (*n* = 562), those who had been diagnosed with LUTS before the end of 2000 (*n* = 1955), and those diagnosed with dementia before the first diagnosis of LUTS (*n* = 344). The date of initial LUTS diagnosis was chosen as the index date. Patients with LUTS who received prescriptions for anticholinergics after the index date were divided into four groups according to their use of anticholinergics. A flow diagram of the sample selection is shown in Fig. 1.

### Outcome

The primary outcome was the occurrence of dementia, defined as a patient who had at least three outpatient visits or one inpatient admission for a principal diagnosis of dementia (ICD-9-CM 290.0–290.4 and 331.0) after the first diagnosis of LUTS. Patients with newly diagnosed dementia within the first year after the index date were excluded. Patients were followed up until development of dementia or until the end of 2012. All the patients were identified in the LHID database to ensure diagnostic accuracy.

### Anticholinergic drug exposure

The primary independent variable of interest was the use of anticholinergics prescribed for patients with LUTS. We lack a gold standard measurement of anticholinergic effect of individual drugs on the human brain. The Anticholinergic Cognitive Burden (ACB) scale was developed in 2008 and updated in 2012. Drugs with higher ACB score [23] are associated with increased risk of cognitive function decline. Drugs with an ACB score of 1 (possibly anticholinergic) have antagonist activity at muscarinic receptors from in vitro data. Drugs with an ACB score of 2 (definitely anticholinergic) have clinical anticholinergic effect identified from literature reviews, prescriber’s information or expert opinion. Drugs with an ACB score of 3 (definitely anticholinergic) not only have clinical anticholinergic effect, but are also associated with delirium from literature reviews, prescriber’s information or expert opinion [23].

In our study, anticholinergic prescriptions were determined by using the Anatomical Therapeutic Chemical system of medications for Flavoxate (G04BD02), Oxybutynin (G04BD04), Propiverine (G04BD06), Tolterodine (G04BD07), Solifenacin (G04BD08), Trospium (G04BD09) from the LHID claims data. All the above drugs were available in Taiwan in treating LUTS and were classified into the ACB score group 3.

The defined daily doses (DDDs) were those recommended by the Collaborating Center for Drug Statistics Methodology of the World Health Organization (WHO) and reflected the assumed average maintenance dose per day for a drug used for its main indication in adults [24]. We used the formula to quantify the use of anticholinergics: (total amount of drug)/(amount of drug in a DDD) = number of DDDs [24]. To measure their risk of increasing dementia, cumulative DDDs (cDDDs) indicating the total exposed dosage of all anticholinergics in ACB score group 3 were estimated as the sum of dispensed DDDs of anticholinergics. We stratified the use of anticholinergics into four levels: < 28, 28–84, 85–336, and ≥ 337 cDDDs.

### Covariates

Covariates were selected as follows: patient’s age at LUTS diagnosis, sex, catastrophic illness certificate, and comorbidities. Age was categorized into five groups: 50–59, 60–69, 70–79, and ≥ 80 years. We used the LHID registry for catastrophic illness certification to identify patients with catastrophic illness, including malignancies and other noncancerous major diseases, such as renal failure, chronic mental disorders, and autoimmune diseases, which were defined by the Bureau of National Health Insurance (NHI). Under Taiwan’s NHI scheme, a certificate of catastrophic illness waives copayment by a patient for medical care, including for outpatient visits and inpatient admissions. Numbers of outpatient visits were defined as the average of annual outpatient visits per person after the index date. Comorbidities were determined by using ICD-9-CM codes as follows: diabetes (250), hypertension (401–405), hyperlipidemia (272), coronary artery disease (CAD; 410–414), cerebrovascular disease (430–438), and atrial fibrillation (427.31).

### Statistical analysis

All statistical analyses were performed using SAS version 9.4 (SAS Institute, Cary, NC, USA). The distribution of patient demographics, catastrophic illness certificates, and comorbidities between the four groups were examined using analysis of variance (ANOVA) for the continuous variables, and Pearson’s chi-squared test or Fisher’s exact test for the categorical variables, where appropriate. The cumulative incidence of dementia by the cDDDs of anticholinergic use was estimated using the Kaplan–Meier method, with comparisons between groups conducted using the log-rank test. We had tested the proportional hazards assumption and the assumption was satisfied [25]. Univariate and multivariable Cox proportional hazard models were then performed to assess the cDDDs of anticholinergic use associated with the risk of subsequent dementia. To check the potential bias of reverse causation, a sensitivity test was performed with multivariable Cox proportional hazard regression analyses by excluding not only the patients diagnosed with dementia within 1 year from the index date, but also within two, three or 4 years to investigate the dementia risk among LUTS patients with different cDDDs. The robust sandwich variance estimator was used to reduce the potential bias of standard errors in misspecification of clustering samples and to increase valid statistical inferences about the corresponding covariate effects [25, 26]. We estimated hazard ratios (HRs) with 95% confidence intervals (CIs). Statistical significance was set at *p* < 0.05 and all tests were two-tailed.

## Results

Characteristics of the participants, and each group categorized by cDDD are shown in Table 1. At the end of December 2005, 16,412 patients newly diagnosed with LUTS were included; the mean age was 66.5 years, and the majority were male. Of these, by the final follow up, 13,681 patients had consumed less than 28 cDDDs of anticholinergic medications, 1285 patients had taken 28–84 cDDDs, 1024 patients had taken 85–336 cDDDs, and 422 patients had received more than 337 cDDDs of anticholinergics. We also analyzed catastrophic illness certificates and other known comorbidities. Patients with LUTS who consumed more cumulative doses of anticholinergics were more likely to be older and have a higher prevalence of diabetes mellitus.

**Table 1:** Characteristics of patients with lower urinary tract symptoms by categories of cumulative defined daily doses of therapeutic bladder anticholinergics

	Total (*n* = 16,412)		< 28 cDDDs (*n* = 13,681)		28–84 cDDDs (*n* = 1285)		85–336 cDDDs (*n* = 1024)		≥337 cDDDs (*n* = 422)		*P*
	n	(%)	n	(%)	n	(%)	n	(%)	n	(%)	
Baseline characteristics											
Age (years), mean (SD)	66.5	(9.6)	66.4	(9.8)	67.1	(8.8)	67.2	(8.5)	67.9	(8.1)	< 0.001
50–59	4346	(26.5)	3787	(27.7)	277	(21.6)	206	(20.1)	76	(18.0)	< 0.001
60–69	5421	(33.0)	4441	(32.4)	447	(34.8)	377	(36.8)	156	(37.0)	
70–79	5192	(31.6)	4173	(30.5)	482	(37.5)	377	(36.8)	160	(37.9)	
≥ 80	1453	(8.9)	1280	(9.4)	79	(6.1)	64	(6.3)	30	(7.1)	
Sex											< 0.001
Male	13,745	(83.7)	11,312	(82.7)	1130	(87.9)	919	(89.7)	384	(91.0)	
Female	2667	(16.3)	2369	(17.3)	155	(12.1)	105	(10.3)	38	(9.0)	
Catastrophic illness certificate	2496	(15.2)	2094	(15.3)	181	(14.1)	142	(13.9)	79	(18.7)	0.078
Comorbidities											
Diabetes mellitus	1093	(6.7)	950	(6.9)	56	(4.5)	57	(5.6)	30	(7.1)	0.002
Hyperlipidemia	236	(1.4)	199	(1.5)	16	(1.3)	15	(1.5)	6	(1.4)	0.946
Hypertension	2019	(12.3)	1706	(12.5)	128	(10.0)	134	(13.1)	51	(12.1)	0.058
CAD	391	(2.4)	323	(2.4)	32	(2.5)	23	(2.3)	13	(3.1)	0.787
CVD	452	(2.8)	392	(2.9)	27	(2.1)	23	(2.3)	10	(2.4)	0.273
Atrial fibrillation	44	(0.3)	37	(0.3)	2	(0.2)	4	(0.4)	1	(0.2)	0.753
Depression	40	(0.2)	33	(0.2)	0	(0.0)	5	(0.5)	2	(0.5)	0.087
TBI	20	(0.1)	19	(0.1)	1	(0.1)	0	(0.0)	0	(0.0)	0.254
Follow-up characteristics											
Number of outpatient visits per year, mean (SD)	27.4	(19.9)	27.1	(19.8)	28.0	(20.2)	28.6	(19.5)	29.8	(20.2)	0.003
Dementia	1666	(10.2)	1372	(10.0)	115	(8.9)	118	(11.5)	61	(14.4)	0.005

*CAD* Coronary artery disease, *cDDDs* Cumulative defined daily doses, *CVD* Cerebrovascular disease, *LUTS* Lower urinary tract symptoms, *SD* Standard deviation, *TBI* Traumatic brain injury.

At the end of the follow-up period, 1666 patients had dementia. The mean (SD) and median (IQR) follow-up time to dementia was 9.0 (2.2) years and 9.2 (8.0–10.7) years respectively for all patients, and were all longer than 9 years in each cDDDs group. Dementia incidence was 10% in the < 28 cDDD group, 8.9% in the 28–84 cDDD group, 11.5% in the 85–336 cDDD group, and 14.4% in the more than 337 cDDD group (*p* = .005). The incidence of dementia was significantly related to higher cumulative doses of anticholinergic use in patients with LUTS. We further analyzed the < 28 cDDD and 28–84 cDDD groups, and found no significant difference between these two groups in dementia incidence (*p* = .326).

In Cox proportional hazards regression analyses (Table 2), the adjusted HR for dementia was 1.15 (95% CI 0.97–1.37) in the 85–336 cDDD group and 1.40 (95% CI 1.12–1.75) in the highest exposure (≥337 cDDDs) group after adjusting for age, sex, catastrophic illness certificates and comorbidities of interest. The sensitivity test of the risk of dementia after excluding those who were diagnosed within 2, 3, or 4 years among patients with LUTS prescribed with different cDDDs is shown in Table 3. We found a trend for adjusted HRs, which increased for the groups with higher cDDDs, when the patients with LUTS who were diagnosed with dementia within 2, 3, or 4 years had been sequentially excluded. In our analysis, higher cumulative doses of anticholinergics manifested as a strong risk factor for dementia in patients with LUTS.

**Table 2:** Cox proportional hazards regression analyses for the risk of dementia among patients with lower urinary tract symptoms and different cumulative defined daily doses of therapeutic bladder anticholinergics

	Univariate model			Multivariate model		
	HR	(95% CI)	*P*	HR	(95% CI)	*P*
cDDD category						
< 28	1.00			1.00		
28–84	0.86	(0.70–1.05)	0.127	0.88	(0.73–1.06)	0.178
85–336	1.11	(0.94–1.32)	0.221	1.15	(0.97–1.37)	0.107
≥ 337	1.39	(1.10–1.76)	0.006	1.40	(1.12–1.75)	0.004
Age (years)	1.08	(1.08–1.09)	< 0.001	1.08	(1.08–1.09)	< 0.001
Sex						
Male	1.00			1.00		
Female	1.16	(1.02–1.31)	0.023	1.35	(1.19–1.53)	< 0.001
Catastrophic illness certificate	1.54	(1.35–1.76)	< 0.001	1.24	(1.09–1.42)	0.001
Comorbidities						
Diabetes mellitus	1.23	(1.04–1.46)	0.018	1.15	(0.96–1.37)	0.121
Hyperlipidemia	1.24	(0.84–1.83)	0.274	1.53	(1.04–2.24)	0.029
Hypertension	1.20	(1.03–1.41)	0.023	0.96	(0.82–1.13)	0.659
CAD	0.99	(0.72–1.36)	0.963	0.72	(0.53–0.97)	0.034
CVD	2.46	(1.89–3.21)	< 0.001	1.92	(1.46–2.52)	< 0.001
Atrial fibrillation	2.21	(1.18–4.12)	0.013	1.41	(0.75–2.63)	0.281
Number of outpatient visits per year	1.01	(1.01–1.02)	< 0.001	1.01	(1.00–1.01)	< 0.001

*CAD* Coronary artery disease, *cDDDs* Cumulative defined daily doses, *CI* Confidence interval, *CVD* Cerebrovascular disease, *HR* Hazard ratio.

**Table 3:** Multivariate Cox proportional hazards regression analyses for the risk of dementia after excluding those who were diagnosed within 2, 3, or 4 years among patients with lower urinary tract symptom prescribed with different cumulative defined daily doses of therapeutic bladder anticholinergics

	Excluding diagnosis within two years^a^				Excluding diagnosis within three years^a^				Excluding diagnosis within four years^a^			
	Events (%)	HR	(95% CI)	*P*	Events (%)	HR	(95% CI)	*P*	Events (%)	HR	(95% CI)	*P*
cDDD category												
< 28	1017 (7.4)	1.00			861 (6.3)	1.00			721 (5.3)	1.00		
28–84	98 (7.6)	1.00	(0.82–1.22)	0.995	92 (7.2)	1.06	(0.86–1.31)	0.568	83 (6.5)	1.15	(0.92–1.43)	0.223
85–336	100 (9.8)	1.30	(1.07–1.59)	0.009	90 (8.8)	1.33	(1.07–1.65)	0.009	77 (7.5)	1.36	(1.08–1.71)	0.008
≥ 337	54 (12.8)	1.69	(1.33–2.15)	< 0.001	50 (11.8)	1.77	(1.39–2.26)	< 0.001	44 (10.4)	1.88	(1.44–2.46)	< 0.001

^a^Adjusted for patient’s age, sex, catastrophic illness certificates, comorbidities, and number of outpatient visits

*cDDDs* Cumulative defined daily doses, *CI* Confidence interval, *HR* Hazard ratio.

**Fig. 2:**
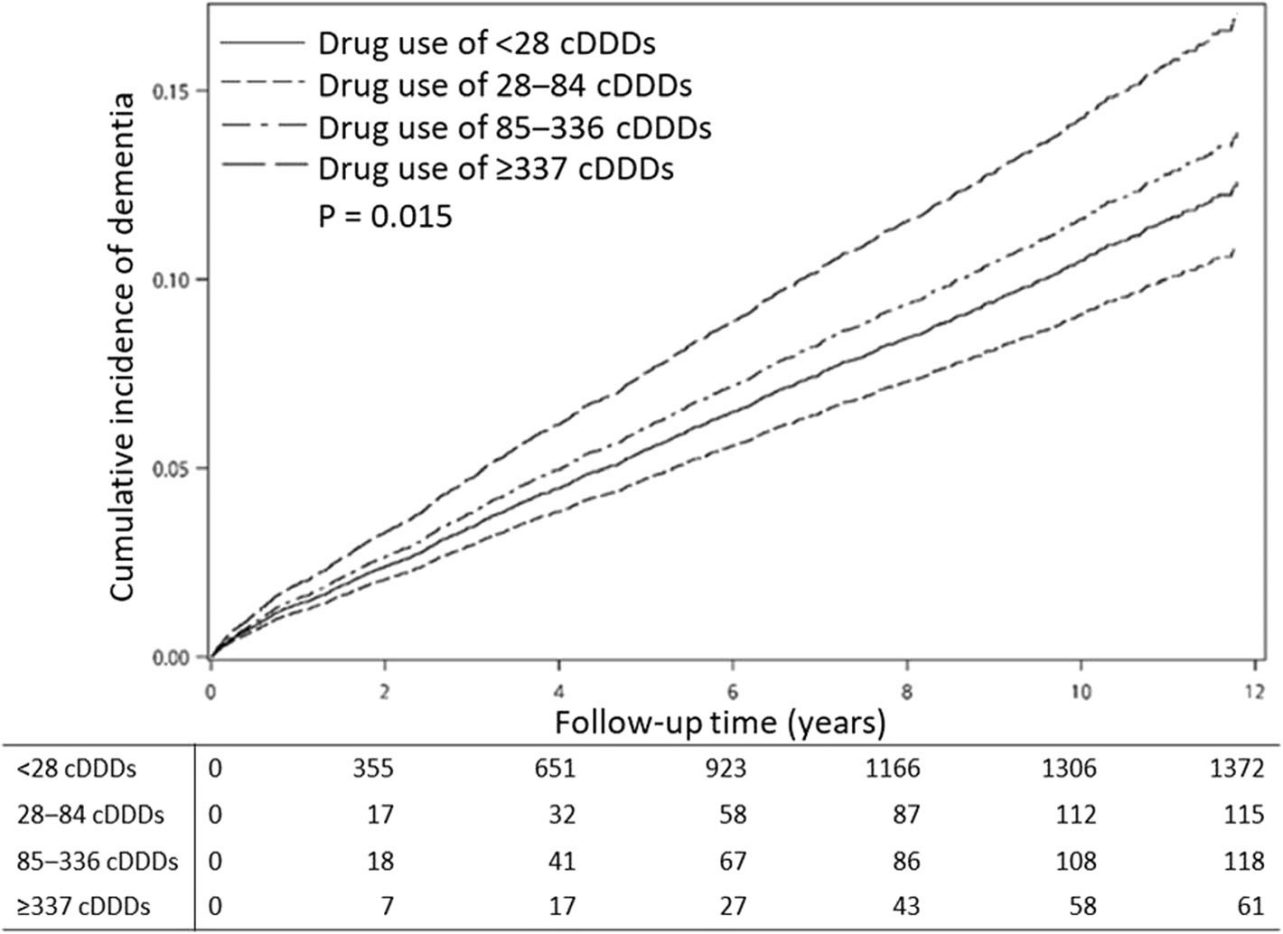
The cumulative incidence of dementia in patients with lower urinary tract symptoms by cumulative defined daily doses of therapeutic bladder anticholinergics

The Kaplan–Meier plot (Fig. 2) with the time / number of dementia cases from the index date showed that there was a significant difference in the incidence of dementia between the four groups of patients with LUTS when classified by cumulative anticholinergic doses (log-rank test *p* = .015). The highest cumulative exposure group, which consumed ≥337 cDDDs, had the highest risk of dementia.

## Discussion

In this nationwide population-based cohort study using data from Taiwan’s NHIRD, we found a significant correlation between cumulative exposure to anticholinergics and the incidence of dementia in patients with LUTS. The HR for dementia in those who consumed ≥337 cDDDs was 1.40 (95% CI 1.12–1.75) compared with those who consumed < 28 cDDDs. To our knowledge, few studies have focused on the dose effect of bladder anticholinergics on the risk of dementia in Asia.

A systematic review analyzing 46 studies found that the use of anticholinergic agents significantly increased the risk of cognitive decline [27]. A large prospective cohort study revealed that higher cumulative use of strong anticholinergics in patients older than 65 years was associated with an increased risk of dementia, and the effect appeared to be lifelong [22].

A recent nested case-control study from general practices in England also concluded that exposure to higher total cumulative doses of anticholinergics was associated with increased risk of dementia in patients older than 55 years, especially in those who consumed more anticholinergic antidepressants, antiparkinson drugs, antipsychotics, bladder antimuscarinic drugs and antiepileptics [28]. Elderly patients using anticholinergics for overactive bladder have been found to have a higher incidence of dementia [29], especially when the anticholinergics have a small molecular size, a neutral charge, are lipophilic, are hydrophobic, and can cross the blood brain barrier easily [30]. The association between LUTS and increased dementia risk was demonstrated in our previous study [17], but the potential contributing factors remain unclear. Bladder anticholinergics are used very widely in the standardized therapy for LUTS, however, little is known about whether this increases the risk of dementia in this patient group or not. We aimed to clinically produce evidence for physicians to weigh-up the benefits and risks of bladder anticholinergics in LUTS treatment, especially in an Asian population. Therefore, we further focused our study on the use of therapeutic bladder anticholinergics only in patients with LUTS in Taiwan. Our findings regarding the cumulative dose effect of bladder anticholinergics echoes the study by Coupland et al. [28] and highlights that this should be considered in clinical practice.

Two randomized controlled trials published in 2005 [31] and 2017 [32] revealed no cognitive decline among elderly patients with LUTS who used Darifenacin or Solifenacin, respectively. These results could be explained by differential selectivity to muscarinic receptors. Among muscarinic receptors, M1-M2 receptors are located in the brain and are important for cognition management, while the M2 and M3 receptors are located in the bladder [30, 33]. If anticholinergics do not selectively block muscarinic receptors, this could increase the risk of cognitive decline. In the two studies above, Darifenacin and Solifenacin are both M3-receptor-selective anticholinergics; therefore, there was no observation of cognitive decline [31, 32]. This difference could be because we focused on the cumulative dose effect of anticholinergic exposure instead of the various properties of each anticholinergic agent. Differences in the selectivity of the M3 muscarinic receptor between each anticholinergic agent should be assessed in future studies.

Conventionally, anticholinergic-induced cognitive impairment appears reversible once the medication has been discontinued [34]. However, there are increasing concerns that the effect might be lifelong [22, 35–37]. A biologically plausible mechanism could be that the cumulative use of these drugs can result in pathological changes similar to those occurring in Alzheimer’s disease [38]. A recent study evaluating structural and functional brain changes in patients who use anticholinergics via structural magnetic resonance imaging, fluorodeoxyglucose F 18 positron emission tomography, and cognitive testing concluded that anticholinergic agents increase whole brain atrophy, reduce glucose metabolism, and cause further clinical decline [21]. Anticholinergics that alter cholinergic pathways are associated with an increased risk of brain atrophy and a decrease in brain function. Reduced cholinergic activity caused by anticholinergics can lead to cell death and neurodegeneration [39–41]. Another hypothesis is that anticholinergics might increase serum corticosterone levels via regulating the hypothalamic-pituitary-adrenal axis, thus promoting neuronal damage in response to stress [42].

Our study has some strengths. First, it was a nationwide cohort study with an adequate follow-up period and a very low attrition rate. Second, although the results of most studies focusing on the long-term use of a single anticholinergic agent have found an increased risk of developing dementia, our results demonstrate that either using one anticholinergic agent or switching anticholinergic agents can cumulatively increase the risk of dementia. Third, although most studies have focused on elderly patients over 65 years, our study included patients starting at 50 years of age, and the increased risk of dementia remained.

Our study does have some limitations. First, there is no gold standard for anticholinergic burden measurement. We focused on the cumulative dose effect of anticholinergic use with incident dementia risk. Second, in this database study, measurement of the exposure relies on the prescriptions filled, and this may not reflect the true amount of medication taken by the patient. Third, common anticholinergics with an ACB score of 3 (definite anticholinergic activity) associated with an increased risk of dementia also include antidepressant, urological and antiparkinson medications [23]. However, we only calculated cumulative doses of bladder anticholinergics available in Taiwan because we wanted to clarify the association of bladder anticholinergics and dementia risk in LUTS patients to inform benefits and risks in clinical practice. Fifth, since LUTS and dementia are both multifactorial disorders, they may share common pathophysiological pathways, that is, LUTS may be symptoms of dementia. Therefore, there is a risk of reverse causation. To explore this, we performed multivariable Cox proportional hazard regression analyses as a sensitivity test, by excluding dementia diagnosis not only within 1 year from index date, but also within 2, 3 or 4 years. We used Cox proportional hazards regression analyses to adjust for important comorbidities and possible confounding factors as far as possible [43]. However, as with many pharmacoepidemiologic studies, we cannot fully account for residual confounding and bias, and factors such as variable prescribing patterns, comorbidities, prognosis and controlling for the time dependency of drug use.

## Conclusions

In this study, we have shown that high cumulative exposure of therapeutic bladder anticholinergics can increase the risk of dementia in people with LUTS. Therefore, anticholinergics should be used with caution in patients with LUTS. Physicians should review and weigh the need for anticholinergics in clinical practice. Behavioral therapy or other medications such as beta-3-agonists could be considered as alternative options. Future work should focus on the properties of various anticholinergic agents, to discover whether they have a differential impact on cognitive function.

## Data Availability

The data that support the findings of this study are available from Taiwan National Health Insurance Research Database but restrictions apply to the availability of these data, which were used under license for the current study, and so are not publicly available. Data are however available from the authors upon reasonable request and with permission of Taiwan National Health Insurance Research Database.

## Abbreviations

ACB: Anticholinergic Cognitive Burden
cDDDs: Cumulative defined daily doses
LHID: Longitudinal Health Insurance Database
LUTS: Lower urinary tract symptoms
NHIRD: National Health Insurance Research Database
